# The neural signature of the Fraser illusion: an explorative EEG study on Fraser-like displays

**DOI:** 10.3389/fnhum.2015.00374

**Published:** 2015-07-02

**Authors:** Xuyan Yun, Simon J. Hazenberg, Richard H. A. H. Jacobs, Jiang Qiu, Rob van Lier

**Affiliations:** ^1^Donders Institute for Brain, Cognition and Behaviour, Radboud UniversityNijmegen, Netherlands; ^2^School of Psychology, Southwest UniversityChongqing, China; ^3^Key Laboratory of Cognition and Personality, Southwest University, Ministry of EducationChongqing, China

**Keywords:** fraser spiral illusion, shape perception, illusion, event-related potentials, complexity

## Abstract

We studied neural correlates accompanying the Fraser spiral illusion. The Fraser spiral illusion consists of twisted cords superimposed on a patchwork background arranged in concentric circles, which is typically perceived as a spiral. We tested four displays: the Fraser spiral illusion and three variants derived from it by orthogonally combining featural properties. In our stimuli, the shape of the cords comprised either concentric circles or a single spiral. The cords themselves consisted of black and white lines in parallel to the contour of the cords (i.e., parallel cords), or oblique line elements (i.e., twisted cords). The displays with twisted cords successfully induced illusory percepts, i.e., circles looked like spirals (the Fraser spiral illusion) and spirals looked like circles (i.e., a “reverse Fraser illusion”). We compared the event-related potentials in a Stimulus (Circle, Spiral) × Percept (Circle, Spiral) design. A significant main effect of Stimulus was found at the posterior scalp in an early component (P220-280) and a significant main effect of Percept was found over the anterior scalp in a later component (P350-450). Although the EEG data suggest stimulus-based processing in the posterior area in an early time window and percept-based processing in the later time window, an overall clear-cut stimulus-percept segregation was not found due to additional interaction effects. Instead, the data, especially in the later time window in the anterior area, point at differential processing for the condition comprising circle shapes but spiral percepts (i.e., the Fraser illusion).

## Introduction

A typical aspect of visual illusions is that the actual percept differs from the presented stimulus. Straight horizontal lines may appear skewed (e.g., the café wall illusion, Gregory and Heard, 1979) or colors may appear at positions that were not exposed to “colored” light (e.g., the neon color illusion, van Tuijl, 1975). Here we use neurophysiological measures to explore differences between illusory appearances and similar but non-illusory appearances. Specifically, we aim to discern neural correlates for the veridical vs. illusory perception of particular shapes.

It is known that a single stimulus may yield very different shape interpretations. In the past decades, rivalry displays and ambiguous stimuli have proven to be excellent materials to study neural correlates of perceptual interpretations. For example, using binocular rivalry in a functional magnetic resonance imaging set-up, differential percept-related cortical activations have been measured given the same presented stimuli (Leopold and Logothetis, 1996). To study the reverse phenomenon in which two different stimuli give rise to the same perceptual interpretation, displays with different figure-ground organizations have been used. For example, Kourtzi and Kanwisher (2001) showed that in a particular area in the ventral pathway (i.e., the LOC), the neural activation did not depend on the particular stimulus properties *per se* but rather on the actual shape interpretation which remained constant despite stimulus change. All in all, such studies have shown dissociations between stimulus-related neural activation and percept-related neural activation.

**Figure 1:**
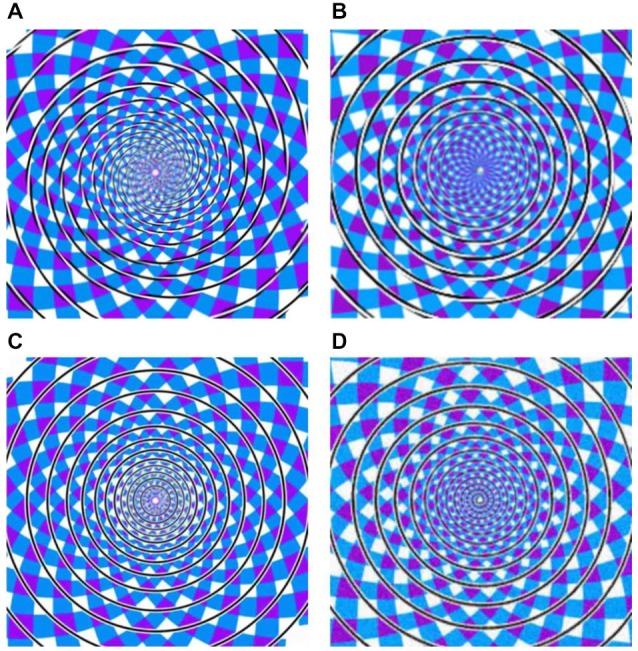
**(A)** Twisted Circles (twisted cords with concentric circles), **(B)** Twisted Spiral (twisted cords with spiral shape), **(C)** the Parallel Circles (parallel cords with concentric circles), **(D)** the Parallel Spiral (parallel cords with spiral shape). Note that these example stimuli differ slightly from the actual stimuli.

In the present study, we focus on such dissociations with regard to one of the best known geometrical illusions, originally described by Fraser (1908). The Fraser spiral illusion (Figure 1A) is made up of a set of concentric circles, against a patchwork background. The circles comprise alternating oblique dark and light parts, which trigger the impression of a twisted cord in spiral shape. In other words, the concentric circles do not appear as circles but as “having a spiral character or tendency” (Fraser, 1908). Previous studies showed that the inclination of the cords is important (Fraser, 1908; Cowan, 1973; Morgan and Moulden, 1986). In 2001, Kitaoka et al. (2001) proposed that there may be special spiral detectors in extrastriate visual cortex integrating the local tilts. The twisted cords would activate these spiral detectors even when the cords are concentric, resulting in the Fraser illusion. Additionally, Pinna and Gregory (2002) reported a similar spiral illusion by using black and white tilted squares. To test the above two cases (same stimulus but different percepts, and different stimuli but same percept), we adopted three variations of the Fraser spiral. We based the construction of these variants on Cowan’s (1973) report that variants of the Fraser illusion may also be hard to categorize as circle or spiral. For the first case, by having alternating dark and light parts in the cords, we constructed a real spiral against the same patchwork background (see Figure 1B for an example). Preliminary observations revealed that this spiral is often seen as a set of concentric circles (i.e., a “reverse Fraser” illusion). In addition, we had two other variants in which the black and white elements in the interior of the lines were replaced by black and white lines in parallel to the outer contours of the circles or the spiral (see Figures 1C,D). In the latter stimuli the illusory shape perception seems much less salient. Given these four displays and the possible percepts triggered by these displays, we have four conditions with regard to stimuli vs. percepts: displays with concentric circles can be seen as a spiral, or as circles, and displays with a spiral can be seen as a set of concentric circles, or as a spiral.

By means of this orthogonal combination of stimulus shape (circle vs. spiral) and perceptual shape (circle vs. spiral), we aim to separate the neural signature of the classical Fraser illusion effect from that of the others by discerning stimulus-related effects and percept-related effects. Note that this design may also differentiate between veridical and illusory percepts (e.g., circle stimulus/circle percept vs. circle stimulus/spiral percept, respectively). In an EEG-setup we asked participants to watch the stimuli and to indicate (by button press) what they actually saw. We expect stimulus related effects to reveal distinctive signals at an earlier time window as compared to percept related effects. In addition, we expect that stimulus-related effects are most likely to be recorded by posterior electrodes.

## Materials and Methods

### Participants

Twenty-four undergraduate volunteers aged 19–23 years from Southwest University (SWU) in China were paid to participate in the experiment. All participants gave written informed consent, were right-handed, had no current or past neurological or psychiatric illness, and had normal or corrected-to-normal vision. This study was approved by the local ethics committee of SWU.

### Stimuli and Procedure

The experiment included four kinds of stimuli: the original Fraser spiral illusion in which a set of concentric circles is made up of twisted cords (referred to as Twisted Circles, see Figure 1A); the reverse Fraser illusion with a single spiral made up of black and white elements (referred to as Twisted Spiral, see Figure 1B); a display with a set of concentric circles made of black and white lines in parallel to the contour (Parallel Circles, see Figure 1C); and a display with a single spiral made of a cord parallel to the contour (Parallel Spiral, see Figure 1D). Note that we did not use the same oblique elements in the reverse Fraser stimulus as in the Fraser displays since that did not result in a strong effect. To obtain the desired effect we simply tweaked the (twisted) cords until the result was satisfactory according to our own preliminary observations (and we additionally tested the percepts behaviorally, see below).

In the actual experiment, the task was to make shape judgments to each display. The participants were seated in front of the monitor. They were instructed to rest their right index finger, middle finger and ring finger on number 1, 2, and 3 of the numeric keypad, respectively. When the picture appeared, they had to indicate the perceived shape by pressing a button as quickly and as accurately as possible (spiral: 1; circle: 2; undecided: 3). In order to check for non-attendance and guessing during the task, a control stimulus was additionally created by scrambling the individual pieces of the display in Figure 1A such that no spiral or circle was present in the display. The frequency of correct response to the control stimulus (i.e., undecided) was exceeding 95%. The pictures were displayed in the center of a 17-inch screen with a 75-Hz refresh rate. The size of the stimuli was 5.5° (horizontal) × 5.5° (vertical). Stimulus order and response hand were counterbalanced across participants.

Before the experiment, participants practiced the task until they reported that they were familiar with the procedure. Since the four stimuli were presented in random order, they were on average shown equally often during practice. The experiment consisted of four blocks, and every block consisted of 75 trials (15 trials per condition including the control-stimuli, randomized). In each trial, the fixation cross appeared for 500 ms, then the stimulus appeared for 1500 ms, after which an empty blank screen appeared for 1000 ms. Participants had to respond during the presentation of the picture or the blank screen. They were instructed to avoid blinking and to avoid making eye movements of any sort and to keep their eyes fixated on the monitor rather than looking down at their fingers during task performance. There was a rest after completing each block. The experiment lasted for approximately 15 min.

### ERP Recording and Analysis

Brain electrical activity was recorded with a sampling rate of 500 Hz using 64 electrodes which were mounted in an elastic cap (Brain Products GmbH, Munich, Germany) and located at the standard positions of the surface of the scalp (International 10/20 system). The ground electrode was placed on the forehead. The EEG was measured with the references on the right and left mastoids, and the signals were re-referenced to the average of the right and left mastoids offline. The horizontal electro oculogram was recorded by two electrodes placed on the outer canthi of both eyes. Eye blinks were monitored with electrodes placed below and above the eye. All interelectrode impedances were kept below 5 kΩ. Signals were filtered with a band-pass of 0.01–100 Hz and a notch filter to remove 50 Hz interference. Ocular correction was performed using the method of Gratton et al. (1983). Signals were offline filtered with a band-pass of 0.01–30 Hz. The ERP waveforms were time-locked to the onset of the stimuli. The averaged epoch was 800 ms, including a 200 ms pre-stimulus baseline. A 200–0 ms baseline correction was applied. Artifacts were removed when the signal exceeded a voltage threshold of ±100 μV.

Based on the behavioral data, using a Stimulus (Circle, Spiral) × Percept (Circle, Spiral) design, there are four conditions: circle shape stimuli with circle percept (Cc), circle shape stimuli with spiral percept (Cs), spiral shape stimuli with circle percept (Sc), and spiral shape stimuli with spiral percept (Ss). That is, the Cc condition includes the trials with circle response to the Twisted Circles and the Parallel Circles. For the other three conditions similar combination of trials could be made. We created ERP waves for these four conditions by averaging the epochs (the mean number of trials used for averaging was 64, 49, 65 and 47 respectively).

Here we chose eighteen electrodes for statistical analysis, nine electrodes from the anterior scalp (F1, Fz, F2, FC1, FCz, FC2, C1, Cz, C2) and another nine electrodes from the posterior scalp (P1, Pz, P2, PO1, POz, PO2, O1, Oz, O2). From observations of the average waveforms, we define the anterior scalp and posterior scalp as our two regions of interest (ROI) in time windows of 160–220 ms, 220–280 ms and 350–450 ms. The amplitude of each ERP component was quantified as the mean voltage within a specified time window, relative to the mean pre-stimulus voltage. For each time window a three-way [ROI (Anterior, Posterior) × Stimulus (Circle, Spiral) × Percept (Circle, Spiral)] repeated measures Analysis of variance (ANOVA) was performed. The p-values corresponding to Greenhouse Geisser method are reported.

## Results

### Behavioral Performance

**Figure 2:**
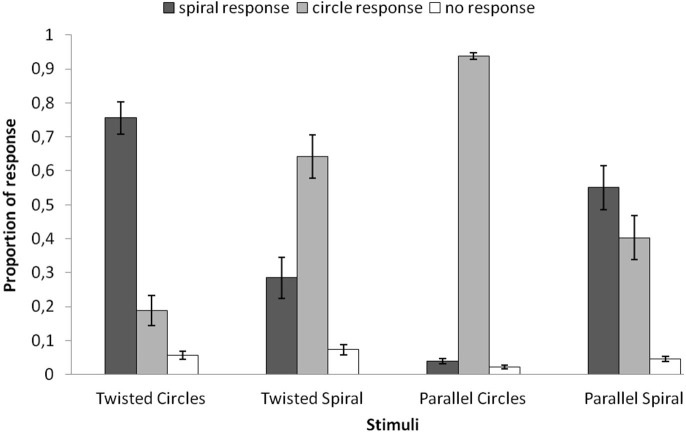
**The average proportion of different responses to the four stimuli.** Spiral responses (dark gray) were made more often in the Twisted Circles than the other three stimuli. The mean proportions of spiral responses were also significantly different between the Twisted Spiral and the Parallel Circles, the Twisted Spiral and the Parallel Spiral, the Parallel Circles and the Parallel Spiral. Error bars represent the standard error of the mean.

In Figure 2 the behavioral results are plotted for each of the four stimuli. For each stimulus the average proportions of the three possible responses are plotted (circle response, spiral response, uncertain/no decision). A repeated-measures ANOVA was applied to the average proportions of spiral response (dark gray bars in Figure 2) with the four stimuli as within subjects factor and showed a significant main effect (*F*_(3,69)_ = 34.951, *p* = 0.000). The average proportion spiral response was highest for the Twisted Circles (*M* = 0.756). This differed significantly from the spiral response to the Twisted Spiral (*M* = 0.285;* F*_(1,23)_ = 39.467, *p* = 0.000), to the Parallel Circles (*M* = 0.040; *F*_(1,23)_ = 200.441, *p* = 0.000) and to the Parallel Spiral (*M* = 0.551; *F*_(1,23)_ = 5.439, *p* = 0.029). Besides, the average proportion of spiral response to the Twisted Spiral was significantly higher than to the Parallel Circles (*F*_(1,23)_ = 16.936, *p* = 0.000), but significantly lower compared to the Parallel Spiral (*F*_(1,23)_ = 7.066, *p* = 0.014). There was also a significantly lower proportion of spiral response to the Parallel Spiral than the Parallel Circles (*F*_(1,23)_ = 65.783, *p* = 0.000).

### Electrophysiological Scalp Data

**Figure 3:**
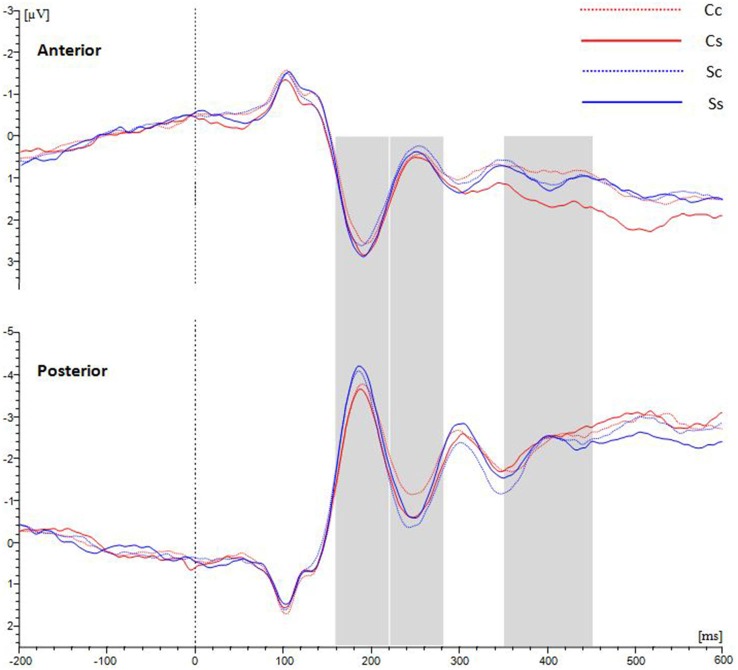
**Grand average ERPs for waveforms distributed in anterior and posterior scalp with average amplitudes for the four conditions.** Between 220–280 ms at the posterior ROI, spiral stimuli (blue lines) elicited a more positive component than circle stimuli (red lines). Between 350–450 ms at the anterior ROI, spiral perception (continuous lines) elicited a more positive component than circle perception (dotted lines).

Those participants for whom the number of available trials was less than 20 in any of the four conditions were eliminated. Ultimately, nineteen observers were included in the ERP analysis. The average waveforms over the anterior scalp and posterior scalp are shown in Figure 3.

**Figure 4:**
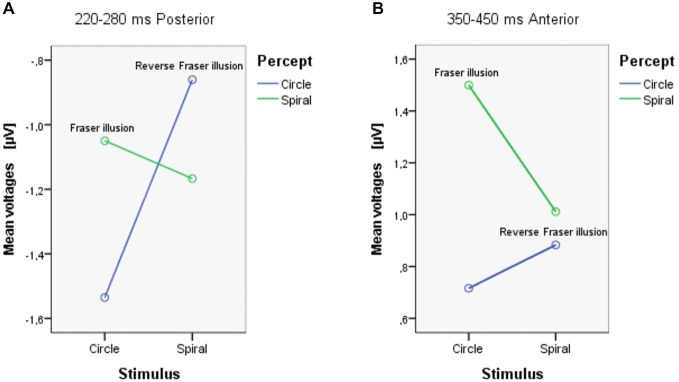
**The mean voltages between 220–280 ms Posterior (A), and between 350–450 ms Anterior (B)**.

In the analysis each ROI (anterior and posterior) was represented by averaging over nine electrodes. Between 220–280 ms, there were marginally significant interactions between Stimulus and Percept (*F*_(1,18)_ = 4.105, *p* = 0.058); ROI and stimulus (*F*_(1,18)_ = 4.148, *p* = 0.057); and ROI, Stimulus and Percept (*F*_(1,18)_ = 3.929, *p* = 0.063). Separate ANOVAs for the anterior and posterior ROIs (see Table 1) show that there was a marginal main effect of Stimulus only in the posterior area (*F*_(1,18)_ = 3.479, *p* = 0.079). There was also a significant interaction between Stimulus and Percept in the posterior area (*F*_(1,18)_ = 8.836, *p* = 0.008; see Figure 4A). Follow-up simple effects test showed that for circle percepts, spiral stimuli (i.e., reverse Fraser illusion generally) elicited a more positive amplitude compared to circle stimuli (i.e., veridical percepts; *p* = 0.004), which is not the case for spiral percepts, when comparing circle stimuli with spiral stimuli (*p* = 0.558). For circle stimuli, spiral percepts (i.e., the Fraser illusion) elicited a more positive amplitude than circle percepts (i.e., veridical percepts; *p* = 0.035), which is not the case for spiral stimuli when comparing circle percepts with spiral percepts (*p* = 0.131).

**Table 1:** **Results: stimulus and percept effects in the anterior and posterior region (9-electrode ROIs)**.

*F (p)*	160–220 ms	220–280 ms	350–450 ms	ANOVA factor
Anterior	0.282 (0.602)	1.827 (0.193)	1.941 (0.181)	Stimulus effect
	2.460 (0.134)	0.956 (0.341)	5.351 (0.033*)	Percept effect
	0.053 (0.821)	0.051 (0.824)	4.583 (0.046*)	Stimulus × Percept
Posterior	2.237 (0.152)	3.479 (0.079)	0.823 (0.376)	Stimulus effect
	0.028 (0.868)	0.339 (0.567)	0.470 (0.502)	Percept effect
	1.688 (0.210)	8.836 (0.008*)	0.042 (0.840)	Stimulus × Percept

*Indicates significance at the 0.05 level.

Between 350–450 ms, there was a significant interaction between ROI and Percept (*F*_(1,18)_ = 4.749, *p* = 0.043). Separate ANOVAs show that there was a main effect of Percept in the anterior area (*F*_(1,18)_ = 5.351, *p* = 0.033) and that there was an additional interaction between Stimulus and Percept in the anterior area (*F*_(1,18)_ = 4.583, *p* = 0.046; see Figure 4B). *Post hoc* tests with regard to the anterior area revealed that the illusory spiral perception (i.e., the Fraser illusion) elicited a more positive amplitude compared to the illusory circle perception (i.e., the reverse Fraser illusion generally; *p* = 0.011), the veridical circle perception (*p* = 0.009) and the veridical spiral perception (*p* = 0.046).

Both Stimulus × Percept interactions should trigger cautiousness with regard to the (marginal) main effects. Notice that this interaction effect can also be driven by a veridical vs. illusion distinction, with illusory percepts (circle stimulus/spiral percept; spiral stimulus/circle percept) resulting in different values from veridical percepts (circle stimulus/circle percept; spiral stimulus/spiral percept). Inspecting Figure 4A, both conditions with illusory percepts indeed evoke the highest voltages. The simple effects, however show that this is only the case for the Fraser illusion. Figure 4B shows a somewhat different result: now one condition clearly distinguishes from the other conditions, i.e., the circle stimulus/spiral percept condition (the Fraser illusion).

## Discussion

The behavioral responses reveal more illusory percepts than veridical percepts for both the twisted circle stimulus (the Fraser spiral illusion) and the twisted spiral stimulus (the reverse Fraser illusion). With regard to the stimuli with the parallel cords the situation was different. The parallel circle stimulus was mainly seen as a set of circles, whereas the parallel spiral turned out to be rather ambiguous. The results suggest that the properties of the cords largely trigger the illusory percept, which is consistent with the previous finding that the illusory effect persisted when the chequered background and the line shape were manipulated (Cowan, 1973).

Next, we used EEG to investigate distinct neural correlates of shape stimuli and percepts. EEG results showed that: (i) over the posterior scalp the amplitude between 220–280 ms tends to be larger for spiral stimuli than for circle stimuli; (ii) over the anterior scalp, the amplitude between 350–450 ms is larger for spiral percepts than for circle percepts; (iii) existing interaction effects preclude a clear-cut stimulus-percept segregation; (iv) simple effects analyses reveal that in both the early and more strongly in the late time window, only for the circle stimuli the voltages for the illusory percepts differed from the veridical percepts.

In the early time window (220–280 ms) the displays that contained spirals elicited a marginally significant stronger component over the posterior scalp as compared to displays that contained circles. Event-related potentials peaking around 200 ms after stimulus onset have been related to the detection of basic features such as brightness, color, and motion (Coch et al., 2005). Besides, the human visual cortex was found to be specialized in many attributes (such as color and motion) of visual stimuli (e.g., Zeki et al., 1991). The stronger component was found only in the posterior area, but not in the anterior area. Hence, the higher amplitude to spiral stimuli may be related to features distinguishing spirals from circles, such as differences in curvature or to complexity differences in general.

An additional effect in the early time window (220–280 ms) is that amplitudes are highest for both illusory displays, although this was significantly different only for the circle stimulus/spiral percept. We speculate that in this time window neural activation is sensitive to conflicts between stimulus and developing percepts. Macknik and Haglund (1999) reported that activation in V1 of rhesus monkeys follows percepts rather than solely the objective stimulus, which may explain why this effect appeared over the posterior region. The posterior higher amplitude might reflect the conflicts between visual scene registration and the illusory perception. Alternatively, another explanation seems plausible here, namely in terms of mere stimulus characteristics since the illusory percepts are predominated by stimuli with twisted cords, while the veridical percepts are predominated by parallel cords. Note also that the circle stimuli perceived as circles mainly comprise displays with parallel cords and therefore can be characterized as the most simple displays revealing the lowest voltages. The spiral stimuli perceived as circles comprise a large number of twisted cord stimuli which can be considered as the most complex stimuli, revealing the highest voltages. All in all, the data trigger cautiousness with regard to effects dealing with the percept or the illusory appearance of the display. To be on the safe side here we support the idea that the data in the early time window may largely be driven by stimulus characteristics of the displays.

The alleged Percept effect in the later time window anterior area at first sight seems to be in agreement with other studies relating late positive waves to perceptual processing. For example, O’Donnell et al. (1988) found a broad positive wave over the frontal cortex during perceptual reversals, as compared to non-reversals in the interpretation of the Necker cube. In addition, Basar-Eroglu et al. (1993) claim that perceptual reversals are associated with a positive wave exhibiting frequency content similar to that of a P300-wave. However, as the Percept effect in the late time window was mainly driven by the responses on the Fraser illusion, it is likely that there is differential processing at this stage between the two conditions with illusory displays in our experiment. One possibility is that the Reverse Fraser display triggers some uncertainty in early stages but may not trigger a sustained illusory percept as is the case with the Fraser display—a difference that could then be reflected in the anterior late time window. Furthermore, following the stimulus complexity driven view on the data in the posterior, early time window, we may conclude that the stimulus characteristics do not play a distinguishable role in the late anterior time window and that it is indeed, the illusory aspect of the Fraser display that has caused the differential results on the Fraser display.

With regard to complexity effects in the early posterior time window we leave the option open that higher order effects like familiarity may account for the difference in activation as well. The spiral shape is not as prevalent as the circle in our natural environment. The early component may reflect the higher familiarity of the circle stimulus (Federmeier and Kutas, 2001) vs. the higher complexity of the spiral stimulus and/or a higher processing demand for the spiral stimulus (Johnson, 1986). With our design, it is not possible to decide between these two possibilities. The results support a view of low brain activity (reflected in closer-to-baseline levels in any measurement, including EEG) for common or relatively expected events, at least at the low-level sensory level (which fits with the Predictive Coding-framework; e.g., de-Wit et al., 2010).

All in all, we may draw the conclusion that the EEG-signature in the 220–280 ms time window posterior is mainly driven by stimulus effects. Although the data do not contradict the view that the differential effects on the Fraser illusion already kick-in in the early time window, a general conclusion with regard to an EEG-signature linked to the percept or illusory appearance would require further testing with different sets of stimuli, also controlling for stimulus complexity and/or familiarity. The 350–450 ms time window reveals differential results for the original Fraser illusion only, and neither to stimuli or percepts *per se*. We suggest that the differential effects may be caused by additional processing due to the conflict between stimulus and percept. The differential signature for the Fraser illusion in this time window may generalize to other illusory displays, but tests with a wider variety of illusory displays would be required to settle the issue of generalizability.

## Conclusion

Our EEG data suggest stimulus based processing in the posterior area in an early time window (220–280 ms). In the anterior area, in a later time window (350–450 ms) we found a differential activation for the condition comprising the Fraser illusion. That is, the EEG signature does not follow a clear-cut stimulus-percept division, but instead points at additional processing triggered by the stimulus-percept conflict in the Fraser illusion.

## Conflict of Interest Statement

The authors declare that the research was conducted in the absence of any commercial or financial relationships that could be construed as a potential conflict of interest.
